# Analysis of the Reduction of Ergonomic Risks through the Implementation of an Automatic Tape Packaging Machine

**DOI:** 10.3390/ijerph192215193

**Published:** 2022-11-17

**Authors:** Ruan C. M. Teixeira, Walter P. S. Guimarães, Josiel G. Ribeiro, Rubens A. Fernandes, Lennon B. F. Nascimento, Israel G. Torné, Fábio S. Cardoso, Gabriella R. Monteiro

**Affiliations:** 1 Embedded Systems Laboratory, State University of Amazonas, Manaus 69050-020, Brazil; 2 Industry 4.0 Laboratory, State University of Amazonas, Manaus 69050-020, Brazil

**Keywords:** industrial automation, ergonomic risks, WMSD, Suzanne Rodgers, REBA, Moore and Garg

## Abstract

Many industrial sectors still lack automation resources to optimize their production processes, aiming to make manufacturing leaner and offer better working conditions to operators. Without these improvements, workers can suffer physical and even psychological damage from the ergonomic risks of the activities performed. Thus, the aim of this paper is to present the ergonomic evaluation of packaging tapes workstation before and after the implementation of an automatic packaging machine, called Guzzetti. In the Guzzetti context, the paper shows the implementation of an electrical system based on controlling a mechanical device powered by servomotors and controlled by a PLC is necessary. For ergonomic evaluation, the paper presents the application of three methods: Suzanne Rodger, Strain Index, called Moore and Garg and REBA (Rapid Entire Body Assessment). With the results collection, was possible to obtain improvements in ergonomic risks that changed from the intermediate level to low level in all methods.

## 1. Introduction

Industrial automation is responsible for technological adaptations in the most diverse production processes. This fact has a significant impact on socio-economic indicators. Automatic system models benefit the industry through the efficient and effective optimization of productive processes. Consequently, the reduction in manufacturing costs and the involvement of labor in the operation of these electromechanical systems grows. In addition, industrial automation offers operators the best working conditions, mainly in activities with too much physical effort that offer ergonomic risk conditions. Electromechanical machinery, for example, can perform repetitive movements over long periods, which can harm the human locomotor system in the short, medium, or long term.

Through these systems, it is possible to mitigate the exposure to the risks of Repetitive Strain Injury (RSI) and Work-Related Musculoskeletal Disorders (WRMD) [1,2]. RSI/WRMD represents diseases that affect tendinous, peripheral nerves, insidious evolution, and musculoskeletal symptoms, usually in the upper limbs [3]. Biomechanical factors that most contribute to the origin of RSI/RT are strength, repetitiveness, frequency, and duration of the activity that can cause tissue overload and increase strains of overexertion, forced labor, and forced labor outsiders [4,5]. As a result, efficient operators working effectively in our industries may experience health problems over time, representing a problem of long-term social and economic efficiency and reduced-term efficiency [6,7].

In the literature, there are tools for evaluating ergonomic risks present in tasks, postures, movement frequency, strength, and muscle use in the workplace. These tools are used in various work environments to assess ergonomic risk factors related to musculoskeletal disorders [8]. The Suzanne Rodgers method is an ergonomic analysis tool for the repetitive movements within the production process. This method evaluates three factors: effort, duration, and frequency of the effort [9,10]. Physical therapists and occupational health professionals apply the Suzanne Rodgers method [11,12].

A relevant method used in the risk assessment of biomechanical overloading of the upper limbs is the Strain Index (S.I.), called the Moore and Garg method. The standard ISO 11228–3:2007 recommends (SI) as a good method for an analytical evaluation [13]. The main objective of the methodology is to evaluate the risk of biomechanical overload related to repetitive movements, taking into account the forces applied by the hands and the postures adopted by the wrists during the work activity [14]. In [15], the SI was applied in the context of blacksmith jobs in Khorda district in Odisha (India) to review the status and discomfort levels of the village craftsmen manufacturing the agricultural appliances.

Another widely used method is the REBA (Rapid Entire Body Assessment). This method provides a joint analysis of the upper limbs, trunk, neck, and lower limbs. In addition, it evaluates the type of grip and the muscular activity performed. Five levels of risk are identified where the lowest scale is called negligible and the highest is called very high [16]. The method is applied in [17] for ergonomic assessment in clothing sector. In addition, the article [18] evaluates the postures assumed by an operator during the manual feeding of a wood-chipper for the assessment of the biomechanical postural overload risk. In [19], the authors applied the REBA to examine risk factors of Musculoskeletal Disorders associated with different work postures during harvesting under variable external conditions.

In order to propose solutions to this problem, the literature presents works in which the use of automatic systems improves the ergonomic aspects of factory operators in many sectors of the industry. In the work [20], for example, the challenges and possibilities for improving ergonomic aspects through cooperative robotics are discussed. On the other hand, the work [21] addresses similar solutions applied in reconfigurable manufacturing systems. Another solution present in literature is the micro break. This method receives this name due to short interruptions of work activities for exercise [22]. The objective of these short pauses is to relieve stress on the musculoskeletal system caused by for unnatural postures [23]. With this, it is possible to perceive the industry’s need for studies to improve health and safety using automation solutions.

Many companies in the tape segment have a high labor force to carry out packaging and manufacturing activities. These activities include allocating labeled tapes in standard-sized cardboard boxes and operating machines at this packaging station. From an ergonomic perspective, this manual activity becomes detrimental to the health of these operators. This harm is due to the high volume of production and the repetitive movement carried out constantly.

This paper aims to present the implementation of an automatic packaging machine model called Guzzetti, developed in an adhesive tape company located in the industrial pole of Manaus, as a way to solve this problem with a system for industrial automation. Section 2 presents the concepts about ergonomics and work-related musculoskeletal disorders and their main symptoms. Section 3 shows the background of ergonomic evaluation methods used in this paper. Section 4 exposes the Brazilian regulamentation for workplace ergonomics. Section 5 shows the materials and methods employed, the architecture of electrical system implemented and the workstation and workers information that was collected in this paper. Section 6 presents the results obtained from the experiments. Lastly, Section 7 and Section 8 shows the discussion and conclusions, respectively.

## 2. Ergonomics and Work-Related Musculoskeletal Disorders (WMSDs)

According to the International Ergonomics Association, IEA, it is possible to divide ergonomics into physical, cognitive, and organizational. In general, this ergonomics corresponds, respectively, to:

1. Posture at work, tool handling, movements performed at work, repetitive movements, and health at work;
2. Mental overload, decision-making and computer interaction;
3. Collective work resources, group projects, cooperative work, organizational culture, telework, and quality management.

It is worth remembering that, according to preliminary data from the National Institute of Social Security (INSS) in Brazil, in 2017, 22,029 accident benefits were granted to workers who needed to be away from work for more than 15 days because of some illness related to repetitive strain injury and work-related musculoskeletal disorder—RSI/WRMD. The number represents 11.19% of all benefits granted. These injuries can occur due to repetitive tasks that require strength or a fast pace of work, added to inadequate postures and stress, forming an environment conducive to the emergence of these injuries or diseases [24]. The main symptoms of work-related musculoskeletal disorders are divided into levels and can be seen in Table 1.

Thus, it is characterized by these lesions and disorders that affect the muscles, tendons, and nerves of the lower members and especially the upper members. It can also affect the neck and trunk. Evolving many times into chronic inflammation and functional consequences [25]. It is worth highlighting that the mentioned concept concerns the characterization of the problems that can occur if there is no ergonomic planning environment for workers. In addition, exposure to the RSI/WRMDs concept is essential for the early identification of problems related to activities that do not have physical ergonomics. However, it is necessary to use methods that indicate whether the works are within the appropriate ergonomic parameters. Some of these methods will be covered in the next section.

## 3. Ergonomic Evaluation

### 3.1. Suzanne Rodgers Method

**Figure 1:**
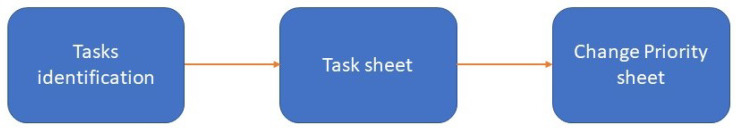
Flowchart of the Suzanne Rodgers method.

The muscle fatigue analysis proposed by Suzanne Rodgers assesses the amount of fatigue accumulated in the muscles during various work patterns with risks of posture and effort during 5 min. Based on the risk of fatigue, a priority for change can be assigned to the task [26]. The implementation follows the flow described in Figure 1.

Using a task identification sheet, the work is divided, then the percentage that each activity represents in the shift and which activities the employee considers difficult is identified. The analysis must be performed for activities with more than 10% of the shift participation. Then, with the work divided into tasks, each task is specified with the risk assessment for each body region. Three risk factors at work are considered, assigning a classification by category. The factors are: level of effort for different organs and regions, duration of continuous effort, and frequency of effort. Finally, when evaluating scores, it is possible to establish priorities for change. It is possible to view an evaluation that reports a priority level, as shown in Table 2, according to the combination of the effort, duration, and frequency categories. The reference tables mentioned above can be found in the work [26]. Based on the combination of the score, it may be to establish the priorities for change, the Table 2 reports the priority level according to the combination of the effort, duration, and frequency.

### 3.2. Rapid Entire Body Assessment Method (REBA)

The REBA method intends to evaluate a broad set of parameters related to biomechanics and human anatomies, such as angles and the relative positions of the limbs and other body parts, characterized as a quick assessment of the whole body [27]. Then, scores are assigned based on specific ranges and threshold values of these biomechanical parameters. REBA extends RULA (Rapid Upper Limb Assessment) by considering the lower body [18]. When applying this method, postures of the upper extremities, lower extremities, trunk, and neck. Each part of the body is assigned a score that will be higher according to the imperatives found. The REBA method also considers the grip’s type or shape and will be more significant the more robust the grip. The maximum total REBA score is 15 and comprises five ranges of values. A risk index and the corresponding recommendations or interventions are established following the different levels of risk found. The risk index can range from negligible to very high, as follows: insignificant risk, no recommended action; low risk, with some changes to consider; medium risk, where some changes are needed; high risk, with changes needed as soon as possible; and very high stakes, where changes must be implemented immediately [28].

### 3.3. Strain Index Method (Moore and Garg)

To measure muscle tension’s repetitiveness and biomechanical aspects, we use the postural Strain Index protocol proposed by Moore and Garg in [29]. This tool makes it possible to quantify the risk in upper limbs by functional overload, simulations of work improvement, and workstation adequacy. The protocol evaluates the effort intensity, effort duration, frequency of movement, wrist and hand posture, work pace, and duration of work per shift. [30,31]. The following are presented the ergonomic evaluation indexes proposed by Moore and Garg.

- Effort intensity: represents the strength needed for a task. It reflects the magnitude of muscular effort required to perform the task. By definition, it is the percentage of maximum force required to perform the task once.
- Duration per effort: is the average time, in seconds, that an effort is applied. A stopwatch can measure the total effort time. To calculate the average duration per effort, one needs to divide the total effort time by the number of efforts counted during the observation period.
- Effort Frequency: is a measure of repetitiveness and is defined as the number of efforts per minute. An analyst or team should observe the task for several complete cycles of work to determine effort per minute.
- Hand/Wrist posture: refers to the anatomical position of the hand/wrist concerning the neutral position. The distinction resides in whether the wrist is in neutral, flexion, or extension. Therefore, analysts must observe the task and determine: whether the wrist is in flexion or extension when applying force and the amount of flexion or extension when applying force. When it is possible to observe different hand/wrist postures, one should use the usual posture that requires the most Maximum Voluntary Contraction (MVC).
- Speed of Work: this is important because of its modifying effects on effort; in other words, the maximum voluntary force decreases, and amplitude increases with increasing speed.
- Duration of Task per day: the total time a task takes in a day. It reflects the adverse effects of prolonged activity, including overtime. Task duration per day measurement unit is in hours.

## 4. The Brazilian Regulamentation for Workplace Ergonomics

In Brazil, the Regulatory Norm 17 (NR 17) of Ergonomics establishes parameters for the adaptation of working conditions and psychophysiological characteristics of workers, providing comfort, safety, and efficient performance for employees [32]. For the evaluation of these adaptations, the employer must perform an ergonomic analysis of the work, called AET, which enables the application of ergonomic concepts to analyze, diagnose, and correct an actual work situation to categorize the activities developed by individuals at work and guide the necessary modifications for a broad adaptation of work modifications, as presented in [33]. This norm suggests using ergonomic analysis tools to perform the AETs, such as those presented in the previous section, considering quantitative and qualitative aspects. In addition, the use of NR-17 guided the implementation of the automation resources used in the design of the Guzzetti machine.

## 5. Materials and Methods

**Figure 2:**
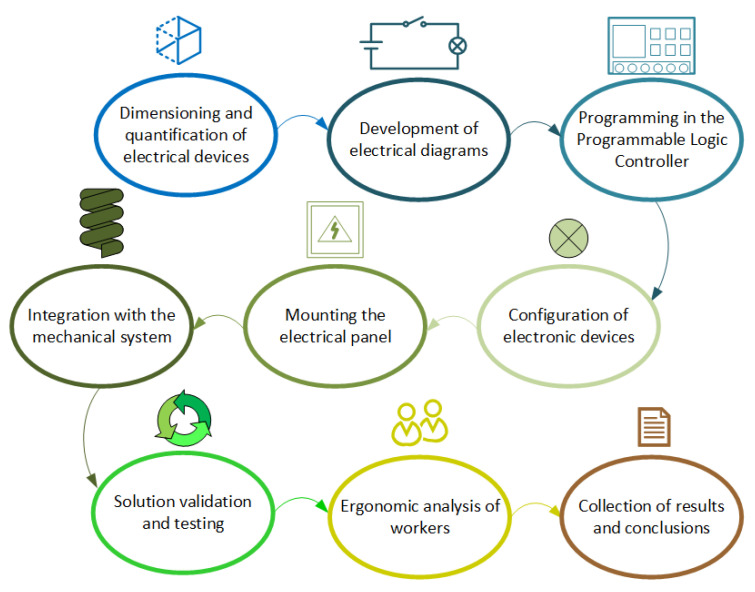
Methodology used for system development.

The mechanical devices and devices that make up the packaging solution were designed in parallel by a team specialized in mechanical engineering and will not be the focus of this work. However, it was necessary to integrate these mechanisms with the electrical system after the development process, and this implementation will be further detailed below. In addition, a methodology for ergonomic analysis of workers before and after the use of machinery was also developed. Therefore, throughout this section, the resources and methodologies necessary to carry out each stage of these processes will be detailed. Figure 2 illustrates a sequential diagram of the steps performed.

### 5.1. Systems for Automatic Packaging of Tape Packages

**Figure 3:**
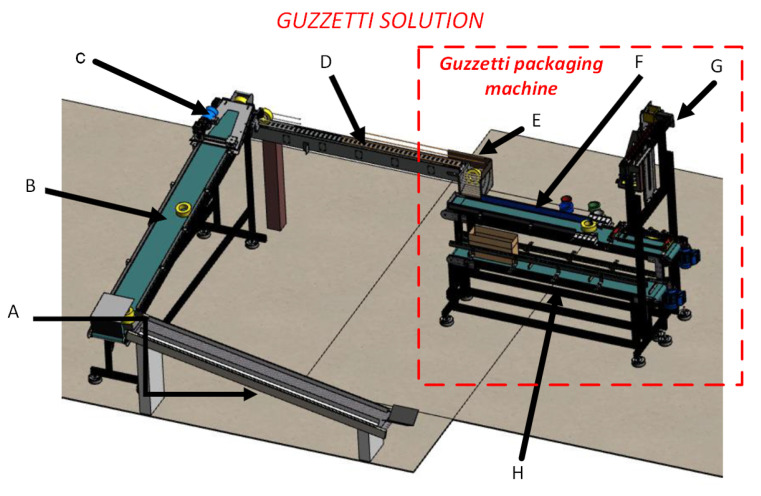
(A) Guzzetti machine exit conveyor; (B) Label conveyor; (C) Labeling machine; (D) Conveyor belt for the tape changer; (E) Tape turner; (F) Conveyor belt Guzzetti; (G) Pick-up system; and (H) Conveyor belt for boxes.

In the case of this project, in particular, the packaging process is an integral part of a set of electromechanical devices responsible for the protection, labeling, and packaging of adhesive tapes in an automatic way known as ”Guzzetti Solution”, which is a patent and owned by the company where this work had been completed. The Guzzetti machine is responsible for inserting a layer of plastic film, used as protection for the adhesive tapes. Figure 3 illustrates this solution and also situates the positioning of the packaging machine in this process.

**Figure 4:**
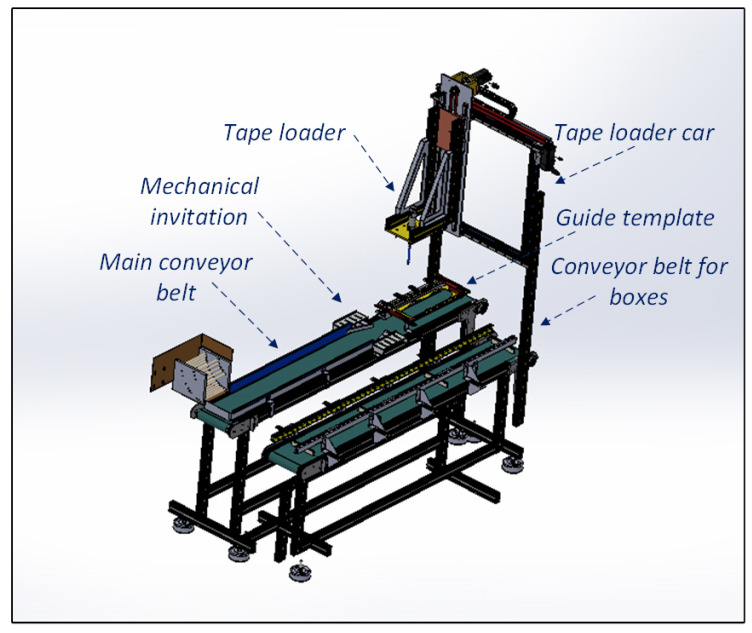
Representation of the packaging machine Guzzetti.

After going through the labeling process, the tapes go through the automatic packaging process, carried out by the Guzzetti packaging machine. This process occurs when allocating tapes in standard-size cardboard boxes located on a conveyor belt parallel to the storage of the tapes. By this step’s end, the box is closed and sealed when the maximum number of tapes is inserted. Figure 4 illustrates the compartments of the Guzzetti packaging machine.

Upon entering the packaging machine, the tapes are in a horizontal position and undergo a mechanical invitation that orders them in a row. Therefore, they enter a guide template that accommodates a certain number of tapes, depending on the model to be packaged. When the last tape enters the template, a photoelectric sensor sends a command to the tape loader to collect the tapes. In this way, it collects all the tapes by displacing them on the “Z” axis, and a system of springs can penetrate the tapes without damaging them so that they can be displaced by the adhesion exerted. The movement system of the tape loader is performed by mechanical devices based on pulleys and belts and using two servo motors: one for movement on the “X” axis and another for the “Z” axis.

**Figure 5:**
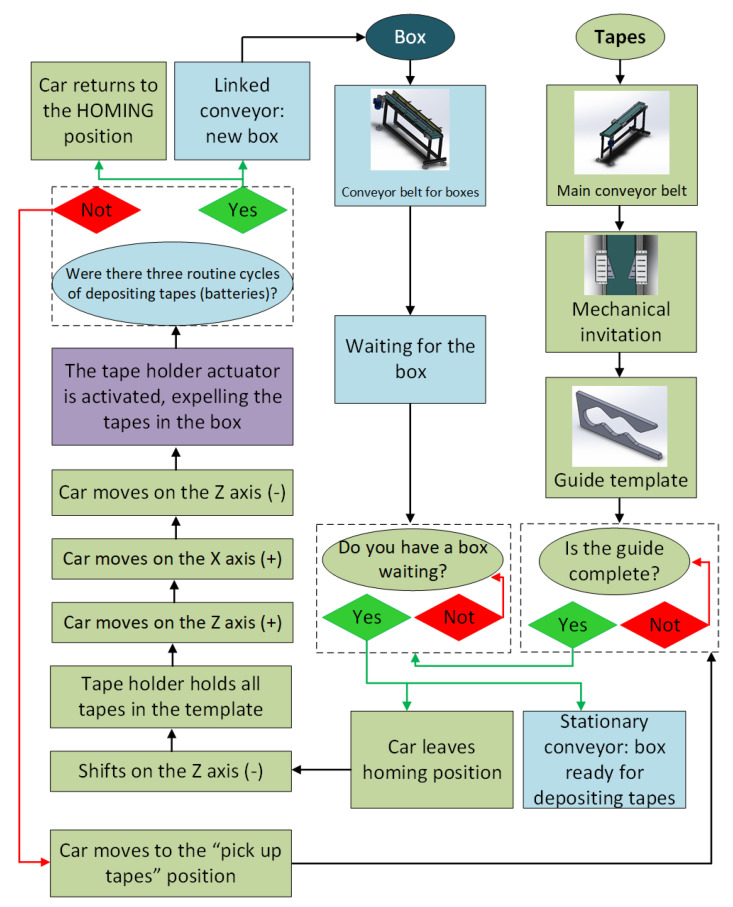
Flowchart of operation of the Guzzetti packaging machine.

The tape loader’s locomotion system is called the “tape loader car.” Its function is to transport the tapes that were in the guide template to the parallel conveyor where the cardboard box is located. Therefore, the tape loader enters the cardboard box. Then, the pneumatic actuator, together with the mechanical solution of the tape extractor, expels the tapes from the loader in the box. After this stage, the loader returns to the starting point, and the process starts again until three stacks of tapes are completed. After the last stack of tapes, the box is complete and can proceed to close. The flowchart of this process is illustrated in Figure 5. It is noteworthy that the home position and the “pick up tapes” position, mentioned in the flowchart, are similar positions when the car is suspended and aligned with the guide. However, the car only departs from the home position in the deposit cycle of the first stack of tapes.

### 5.2. Electrical System Architecture

The central control element of the Guzzetti packaging machine is the programmable logic controller (PLC). Therefore, it is possible to read sensors and switches generally and control physical peripherals, such as frequency inverters, servo motor drivers, signaling devices, and pneumatic actuators.

As external input devices for the system, push-buttons were used for the start/stop function and restarting the system, if necessary, in addition to using an emergency key to stop the entire process. A limit switch sensor was also implemented in the application to signal to the PLC the limit of the tape loader displacement, which, in turn, will respond to the servo-motor driver the commands to end the cycle. It was necessary to use four photoelectric sensors, where two of them indicate when the template is complete, aligned with the last and first position of the template, and the other two indicate when the box is prepared and positioned to receive the tapes.

**Figure 6:**
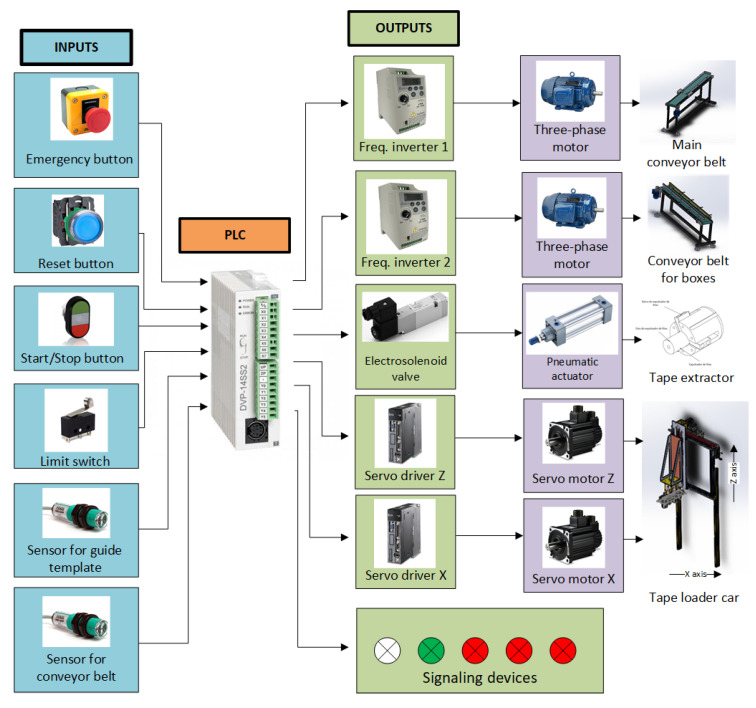
Architectural diagram of the electrical system.

At the output of the PLC, two frequency inverters were used to control the speed and direction of each used conveyor. The servos control was carried out through the communication of the PLC with their respective drivers, and they will act in the movement of the loading car and the “pick-up” system. The PLC also controls the electro-pneumatic drive of the tape extractor. Signals were used to indicate energization, overloads, and sensor failures and whether the system was on or off. The architectural diagram of the electrical system is illustrated in Figure 6.

### 5.3. Implementation of the Electromechanical System

The Guzzeti machine’s implementation integrates electromechanical components that make up the system. Thus, it is possible to divide this process into the assembly of the control panel and the integration of mechanical components. After completing the electrical diagram, it was possible to start assembling the electrical panel following the planned connections. For that, a layout of the components, channels, and rails for mobility and allocation was created to assemble the control panel, favoring the entire primary infrastructure. Then, with the previous positioning of the components, the panel cabling process started. The cabling was classified according to its purposes: power and control circuits. For the dimensioning of the power circuit cables, the minimum session 2.5 mm criterion was used according to the specified current, and for the control circuit, cables of 1 mm were used. From this, it was necessary to adapt the outer part of the cabinet for coupling the buttons, signs, disconnect switch, and cable gland and their appropriate connections.

**Figure 7:**
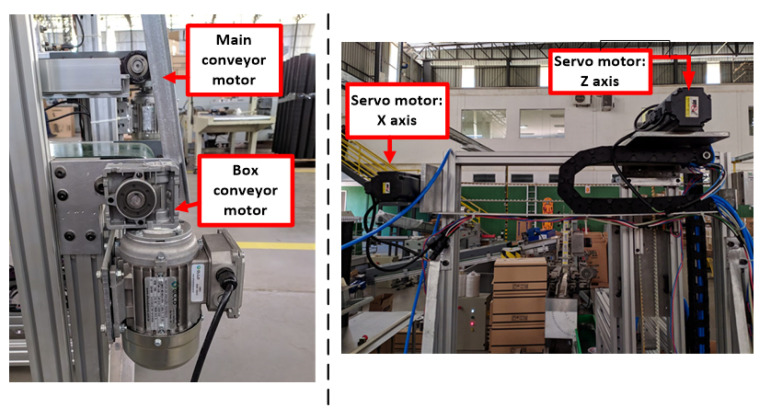
Installation of three-phase motor and servo-motors.

**Figure 8:**
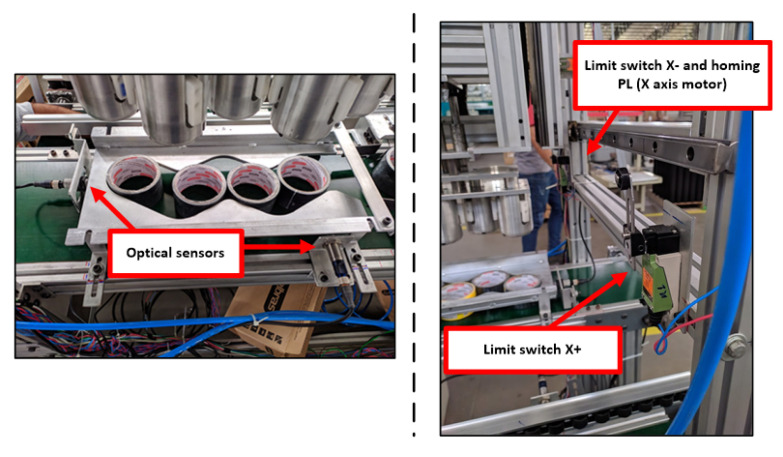
Installation of optical sensors and limit switches.

**Figure 9:**
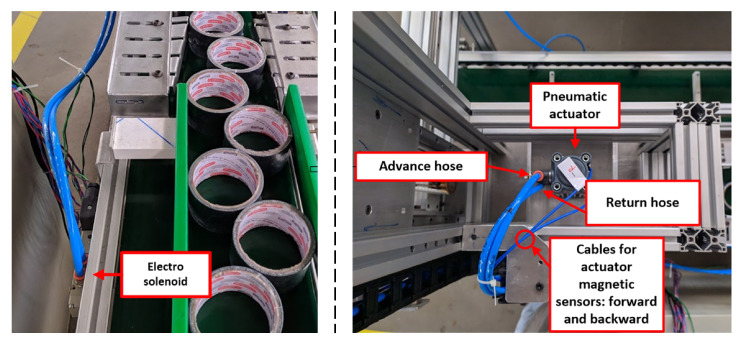
Pneumatic circuit installation.

**Figure 10:**
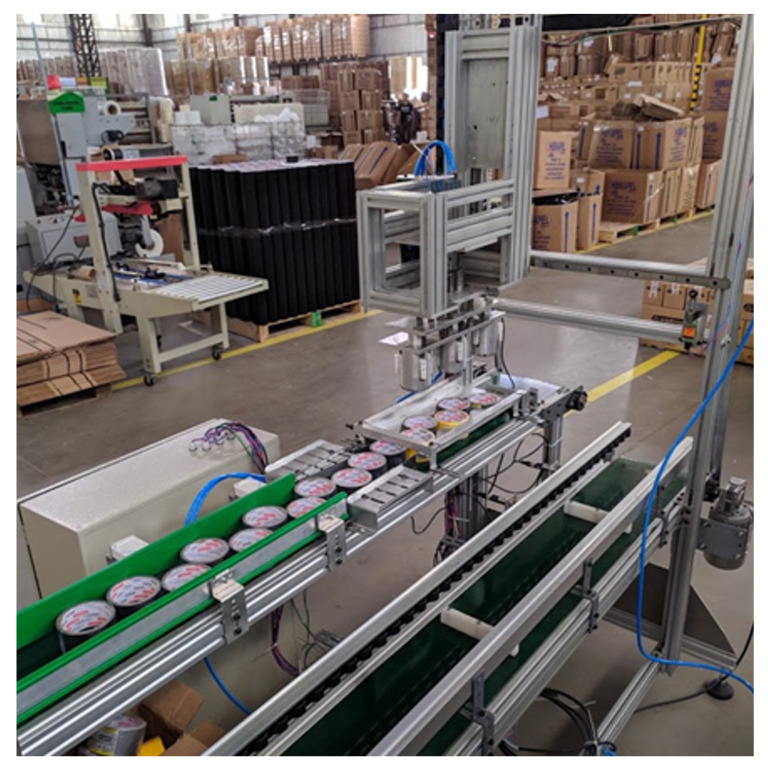
Guzzetti machine installed.

After completing the control panel assembly, it was possible to integrate with the mechanical solution. The peripherals allocated in the mechanical part were the servo-motors, three-phase induction motors, optical sensors, limit switches, and the pneumatic system. The servo motors were coupled to the toothed pulley of the tape loader carriage that uses the principle of belt and pulley to carry out the displacement of the carriage on the x-axis and z-axis and the integration of three-phase induction motors on the track axis, the track motors as shown in Figure 7. The next step is the allocation of sensors, starting with the two sensors that detect when the guide template is complete, and the loader carriage can now start its deposit routine. In addition, the limit switch must be installed on the mechanical structure at the end of the track loader carriage track to signal the limit of the servo-motor operation and indicate the homing position. These elements can be seen in Figure 8. Finally, the pneumatic system, consisting of integrating the pneumatic actuator with the solenoid valve and the pneumatic circuit, was installed. Figure 9 shows the output of the sensor cables, which indicates the status of the pneumatic actuator, in addition to allowing advance and return commands for compressed air through a valve. After the entire implementation process, it is possible to obtain the entire electrical system structured in the Guzzeti packaging machine, as shown in Figure 10.

### 5.4. Organizational Information

A search for information on the workstation and the operators involved must be carried out before the ergonomic analysis of the activity, in accordance with Regulatory Norm 17 [32]. Table 3 shows the characteristics of the workstation and the operator involved in the tape wrapping activity at the time of analysis. These data were obtained through interviews with the production manager and operators of the packing station and were used to carry out the AET. It is important to highlight that, in addition to the reduction in ergonomic risks alerted by the company’s work safety team, the operator complained of WMSDs, level 1. Thus, this was one of the motivations for implementing the packaging machine presented in our work.

## 6. Results

This section presents the results collected from the analyzes carried out with the ergonomic evaluation methods, before and after the automation resources inserted with the Guzzetti packaging machine.

### 6.1. Analysis before Automation

**Figure 11:**
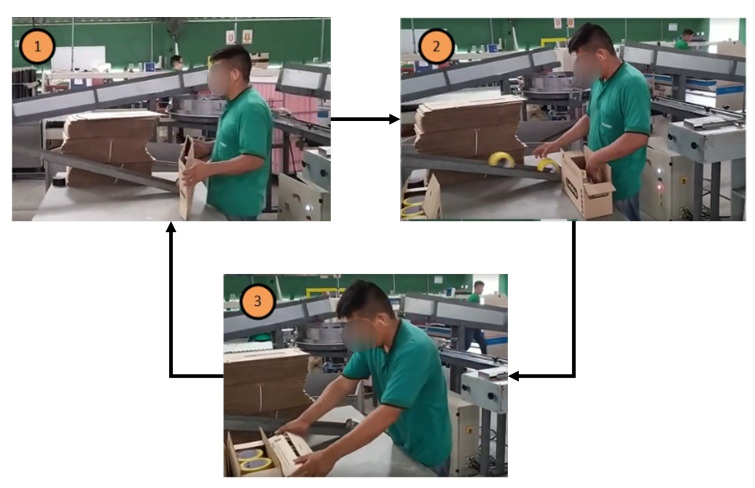
Manual adhesive tape packaging operation.

It was necessary to understand the functioning of the tape packaging before the solution to highlight the impact of the Guzzetti packaging machine results. First, the operator had cardboard boxes stacked on the side of the ramp, from where he received the tapes at the workstation. Then, the tapes were placed in standard cardboard boxes. After completing each box, the operator would move it to the left to complete the packing process and start another cycle. Figure 11 illustrates this manual procedure, including the three sequential phases of this process. In terms of biomechanics, step 1 of the figure illustrates the operator’s elbow flexion. Step 2 exposes cervical flexion, elbow flexion, right wrist flexion, and right palm grip. Finally, step 3 illustrates cervical flexion, elbow flexion, and bilateral palm grip.

However, in ergonomic terms, the activity had some aggravating factors. Above all, in the frequency and duration of the efforts of this activity, as there were no intervals between the packaging cycles, it was necessary to carry out movements with the fingers to pick up all the production tapes and deposit them in their respective boxes. Thus, applying ergonomic evaluation based on Suzanne Rodgers and Moore and Garg methods can be seen in Table 4 and Table 5, respectively. In addition, the REBA results can be seen in Table 6 and Table 7.

### 6.2. Analysis after Automation

**Figure 12:**
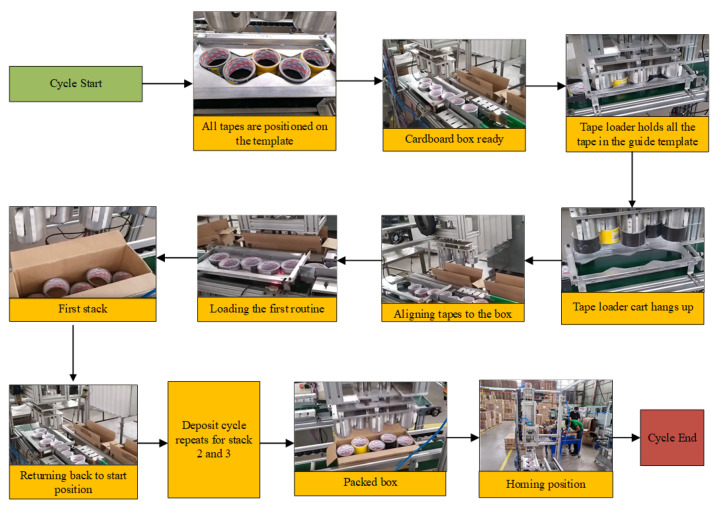
Routine cycle of the Guzzetti packaging machine.

After installing and using the machine, it was possible to package the tapes automatically, as proposed in this paper. All the main routines of the Guzzetti wrapper are in Figure 12.

**Figure 13:**
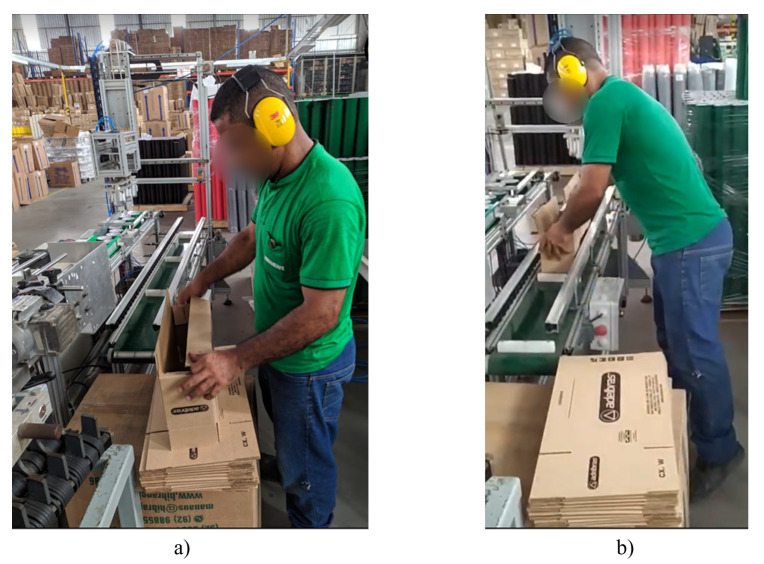
Box assembly (**a**) and positioning operation (**b**).

However, manual activity is still required after the installation of the machinery. The activity consists of assembling the box (a) and positioning it on the conveyor (b), as shown in Figure 13. In terms of biomechanics: step 1 of the figure illustrates cervical flexion, elbow flexion, and bilateral palmar grip; step 2 exposes cervical flexion, shoulder flexion, elbow flexion, and bilateral palm grip. Although, this movement requires much less effort. This fact can be evidenced by performing the ergonomic analysis after automation. The results of Suzanne Rodgers and Moore and Garg Evaluation are shown in the Table 8 and Table 9. The REBA results after the automation process are shown in Table 10 and Table 11.

It was observed that the implementation of the proposed solution automated the tape packaging station to replace the manual operation with machinery capable of transporting three stacks of tapes sequentially to standardized cardboard boxes. However, there is still a need for an operator to carry out the activity of assembling the box and depositing them on the conveyor. However, this activity has a frequency 93% lower than the manual tape packaging operation, and the interval between cycles went from 0 to 14s, resulting in two significant improvements.

The first improvement is optimizing the ergonomics of the packaging activity since there are fewer repetitions per minute. This evidence was observed in the ergonomic analyses following the model of Suzanne Rodgers, where the manual packaging activity had three priorities of moderate change and one high. Before automation, the Moore and Garg results show two tasks with low risk and one task with moderate risk as presented in Table 5. The REBA result, according to Table 7, ratifies moderate risk. After automation, all ergonomic evaluation methods showed the improvements achieved by decreasing the risk from moderate to low.

The second improvement was observed in the interval between cycles because the employee could operate other machines at his post during this interval. Previously, two operators were needed for the packing station. With the advent of the Guzzetti packaging machine, only one operator can carry out both activities, making manufacturing leaner. In turn, the cycle time relating manual operation to automatic operation was another production data analyzed. The cycle time with manual operation was 19 s, while the cycle time with the Guzzetti machine was 23 s.

Despite ergonomic improvements and manufacturing optimization, there was an increase in cycle time with the advent of the station automation. This is due to the rise in the servo-motor routines time, aiming at the movement precision and tape quality maintenance. However, this fact did not prove harmful to the company since the packing station has a shorter cycle time than the other stations. Therefore, this allows a margin for increasing the cycle time.

## 7. Discussion

Notably, through the automation of the packaging station, it is possible to stress two highly relevant improvements for the employee’s health since a non-automated manual process generates much more fatigue. Fatigue is one of the leading causes of reduced industrial productivity. In some cases, it is relatively easy to locate the sources of fatigue, which may be excessive muscular load or unfavorable environments. In other cases, they relate to schedules, shift work, production scheduling, or social relationships inside and outside work [34]. Therefore, by performing an ergonomic evaluation with Suzanne Rodgers, Moore and Garg, and REBA before and after the automation process, it was possible to identify the aforementioned improvements.

Another significant impact of the Guzzetti packaging machine results was micro-breaks during work cycles, according seen in Table 12. They perform as a second type of spontaneous break during the working day and occur when the worker has a rest time, even if minimal, between the activities executed, so when the worker starts the work of a part, concludes, and when will start a new one, take a short rest interval. Such work breaks can occur when they are tired, fatigued, or feel some need to stop. Although they are essential for the worker’s rest and recovery of his physical state, minimizing the risks [35].

## 8. Conclusions

Regarding the development of the electrical system for the automatic solution, it was possible to conclude that the proposed methodology proved to be adequate. This statement is supported by the good performance of the machinery in all proposed functionality and reliability tests. In addition, the entire tape packaging process has become fully autonomous. Therefore, the machine was able to work in a high-volume production regime. The automatic tape packaging system showed satisfactory results in improving the working conditions of the tape packaging station operator. Because the worker was relocated from a manual packaging activity to an activity with fewer cycles and, consequently, less harmful to human physiology, this can be proven by comparing the ergonomic analysis of the Suzanne Rodgers, REBA, and Moore and Garg methods, that after the change, showed a significant attenuation in the priority change of the new work routine. Therefore, from the results obtained with the automation of the productive environment and improvements in the operator’s working conditions, it is concluded that the proposed implementation contributed significantly to the ergonomic conditions of the packaging production process.

## Figures and Tables

**Table 1:** WMSD—Main symptoms.

Levels	Description
Level 1	Discomfort sensation and localized pain
	in the affected limb. Usually mild and
	intermittent, improves with rest;
Level 2	Tolerable pain, however more persistent,
	intense and localized. The recovery can
	be achieved by rest, but it takes longer
	than level 1;
Level 3	Persistent and severe pain, reduced
	muscle in strength, loss of control of
	movements, variation sensitivity, does
	not attenuate with rest;
Level 4	Severe (unbearable) pain, radiated to all
	affected segments, inability to perform
	the tasks and may have deformities and
	atrophies.

**Table 2:** Scores for priority levels.

Low (L)	Moderate (M)	High (H)	Very High (VH)
111	123	223	323
112	132	313	331
113	213	321	332
211	222	322	4xx, x4x, xx4 *
121	231		
212	232		
311	312		
122			
131			
221			

* A level 4 category for effort level, continuous effort; duration or frequency is automatically Very High (VH).

**Table 3:** Characterization parameters of the job and labors.

Parameters	Answers
Function	Production operator
Operator Age	30–35 years
Operator Height	169–173 cm
Department	Packing
Task type	Cyclic
Volume per shift	720 boxes
Relay Station	Yes
Shift	1st
Lunch time	60 min
Break	10 min
Actual hours worked	410 min

**Table 4:** Suzanne Rodgers analysis before automation process.

Region	Side	Effort Level	Effort Duration	Effort Freq.	Priority
Shoulder	Right	2	2	2	Moder.
	Left	1	2	2	Low
Back	-	2	1	2	Low
Arms/Elbow	Right	2	2	2	Moder.
	Left	1	2	2	Low
Hand/Finger	Right	2	2	3	High
	Left	2	2	3	Moder.

**Table 5:** Moore and Garg analysis before automation process.

Task	Box Assembly	Manual Tape Placement	Box Closing
Effort intensity	1	1	1
Duration per effort	1	2	1
Effort Frequency	0.5	2	0.5
Hand/Wrist posture	1.5	1.5	1.5
Speed of Work	1	1	1
Duração do Trabalho (FDT)	1	1	1
M&G Index [29]	0.75	6	0.75

**Table 6:** REBA group scoring before automation.

Group [27]	Moviment	Score
A	Trunk	2
A	Neck	2
A	Legs	2
B	Arms	3
B	Forearm	1
B	Wrist	3

**Table 7:** REBA score before automation.

Table C [27]	Activity Score	REBA Score	Scoring Description
6	1	7	Medium risk (change soon)

**Table 8:** Suzanne Rodgers analysis after automation process.

Region	Side	Effort Level	Effort Duration	Effort Freq.	Priority
Shoulder	Right	2	1	2	Low
	Left	1	1	2	Low
Back	-	2	1	2	Low
Arms/Elbow	Right	1	2	2	Low
	Left	1	2	2	Low
Hand/Finger	Right	2	1	2	Low
	Left	2	1	2	Low

**Table 9:** Moore and Garg analysis after automation process.

Task	Box Assembly
Effort intensity	1
Duration per effort	1
Effort Frequency	0.5
Hand/Wrist posture	1.5
Speed of Work	1
Duração do Trabalho (FDT)	1
M&G Index [29]	0.75

**Table 10:** REBA group scoring after automation.

Group [27]	Moviment	Score
A	Trunk	2
A	Neck	2
A	Legs	1
B	Arms	2
B	Forearm	1
B	Wrist	2

**Table 11:** REBA score after automation.

Table C [27]	Activity Score	REBA Score	Scoring Description
3	0	3	Low risk (change may be necessary)

**Table 12:** Micro break occupancy rate analysis.

Operations	Frequency	Micro Break (s)	Occupancy Rate
Before automation	720	0	55.61%
After automation	720	14	67.32%

## Data Availability

The data analyzed during the current study are available from the corresponding author on reasonable request.
